# ﻿Phylogenetic analysis of the Neotropical scarab beetle tribe Aegidiini (Coleoptera, Scarabaeidae, Orphninae) with description of new taxa

**DOI:** 10.3897/zookeys.1166.102813

**Published:** 2023-06-06

**Authors:** Andrey V. Frolov, Lilia A. Akhmetova, Jhon César Neita-Moreno

**Affiliations:** 1 Laboratory of Insect Systematics, Zoological Institute, Russian Academy of Sciences, Universitetskaya nab., 1, St.-Petersburg 199034, Russia Zoological Institute, Russian Academy of Sciences St. Petersburg Russia; 2 Instituto de Investigaciones de Recursos Biológicos Alexander von Humboldt, Claustro de San Agustín, Boyacá, Colombia Instituto de Investigaciones de Recursos Biológicos Alexander von Humboldt Boyacá Colombia

**Keywords:** Caqueta moist forests, Colombia, new species, new subtribes, orphnines, Peru, Peruvian Yungas, phylogeny, scarabs

## Abstract

In the Neotropics, orphnine scarab beetles are represented by the endemic tribe Aegidiini Paulian, 1984 with five genera and over 50 species. Phylogenetic analysis based on morphological characters of all supraspecific taxa of Orphninae showed that Aegidiini is comprised of two lineages. New subtribes, Aegidiina subtr. nov. (*Aegidium* Westwood, 1845, *Paraegidium* Vulcano et al., 1966, *Aegidiellus* Paulian, 1984, and *Onorius* Frolov & Vaz-de-Mello, 2015, and Aegidinina**subtr. nov.** (*Aegidinus* Arrow, 1904) are proposed to better reflect this phylogeny. Two new species of *Aegidinus* are described: *A.alexanderi***sp. nov.**, from the Yungas in Peru and *A.elbae***sp. nov.** from the Caqueta moist forests ecoregion in Colombia. A diagnostic key to *Aegidinus* species is given.

## ﻿Introduction

The scarab beetles of the subfamily Orphninae are distributed mostly in the tropics of the southern continents. In the Neotropics, they are represented by the endemic tribe Aegidiini Paulian and comprise five genera and over 50 species (Paulian 1984; Colby 2009; Frolov and Vaz-de-Mello 2015; Frolov et al. 2017a, b, c; Rojkoff and Frolov 2017; Frolov et al. 2019). In the previous phylogenetic analysis of the Orphninae (Frolov 2012), some characters were misinterpreted due to the limited material then available. After this preliminary analysis was published, a new genus of the South American Orphninae was also described (Frolov and Vaz-de-Mello 2015). The aim of the present work, apart from the description of a new species of *Aegidinus* Arrow, is to provide the results of the phylogenetic analysis of the tribe Aegidiini based on a verified and expanded set of morphological characters of all nominal supraspecific taxa of the Aegidiini and make the classification better reflect the phylogenetic relations of the taxa in question by introducing a subtribal level with two new taxa.

The genus *Aegidinus* currently comprises 14 species distributed in South America, mostly in the Amazon and Guiana moist forest regions to the Yungas in the west, and in Trinidad Island (Colby 2009; Frolov et al. 2019). Recently we had an opportunity to examine two series of *Aegidinus* specimens from south-eastern Peru. The series included both males and females and are similar to *A.teamscaraborum* Colby, 2009, yet the males have a different shape of the parameres. Below the new species is described.

## ﻿Material and method

The material used in this work is housed in the collection of the Zoological Institute, Russian Academy of Sciences, Saint-Petersburg, Russia (**ZIN**), Instituto Alexander von Humboldt, Villa de Leyva, Boyacá, Colombia (IAvH), and Canadian Museum of Nature, Ottawa, Canada (**CMN**). Morphological terminology follows Frolov (2012) and Frolov et al. (2016). In the new species descriptions, labels of the type specimens are cited verbatim and separated by a slash and our comments are in square brackets. Preparation of specimens, digital images and locality maps follow Akhmetova and Frolov (2022).

The maximum parsimony (MP) analyses were conducted in TNT 1.6 (Goloboff and Morales 2023) using the “traditional search” option to find the most parsimonious trees (MPTs). The following parameters were used: memory set to hold 1 000 000 trees; tree bisection–reconnection (TBR) branch-swapping algorithm with 1000 replications saving ten trees per replicate; zero-length branches collapsed after the search. All character states were treated as unordered and equally weighted. Autapomorphic characters were deactivated before the parsimony analysis. Bremer support was calculated using the TNT Bremer function, using suboptimal trees up to 20 steps longer. For character mapping, Winclada v.1.00.08 (Nixon 2002) was used with unambiguous optimization.

## ﻿Results

### ﻿Phylogenetic analysis of the Aegidiini

Ingroup and outgroup

In the ingroup, we included all generic taxa of the Orphninae with the following exceptions. The monotypic genera *Hybaloides* Quedenfeldt, 1884 and *Craniorphnus* Kolbe, 1895, known from single type specimens, are not included since they are based on misidentified *Orphnus* species (unpublished data of the authors). The two genera, *Onorius* Frolov & Vaz-de-Mello, recently described from the Andes (Frolov and Vaz-de-Mello 2015), and the central African *Cerhomalus* Quedenfeldt, 1884, restored as a genus distinct from *Orphnus* (Frolov and Akhmetova 2021), are added to the list of ingroup taxa used by Frolov (2012). From the outgroup, we excluded the distantly related taxa of the family Hybosoridae and included the two species of the genus *Allidiostoma* Arrow, 1904. The latter is a member of the small, olygotypic subfamily Allidiostomatinae Arrow, 1904, distributed in southern South America (Ocampo and Colby 2009). There is evidence that Allidiostomatinae might be a sister group of the Orphninae (Ocampo and Hawks 2006; Ocampo et al. 2010; Neita‐Moreno et al. 2019).

#### ﻿Character states and their codes

The character states are based on the previous phylogenetic analysis (Frolov 2012) with the following modifications:

##### Characters excluded

The characters 14, 17, 37, 41, 42 (Frolov 2012) were excluded because they become uninformative after Hybosoridae was excluded from the analysis. Characters 24 and 25 are excluded since they are related to flightlessness which occurs in many Orphninae taxa and apparently evolved many times in different lineages (Frolov and Akhmetova 2020). Character 31 is excluded because it is a character of sexual dimorphism found in different non-related taxa (i.e., Scarabaeinae dung beetles of the genera *Macroderes* Westwood, 1842 and *Xinidium* Harold, 1869).

##### Characters added

Parameres: symmetrical (0), asymmetrical (1).

Stridulatory ridges: straight (0), distinctly curved posteriad (1).

Phallobase protruding ventroapical plate: absent (0), present (1).

Mediobasal margins of parameres: feebly sclerotised (0), strongly sclerotised (1), strongly sclerotised and serrate (2).

Mandibles visible from above: yes (0), no or feebly (1).

Labrum visible from above: yes (0), no (1).

Tarsi: slender (0), robust (1).

Paramere apices: glabrous (0), with short setation (1), with long setation (2).

Tubercle on anterior margin of pronotum in female: absent (0), present (1).

Clypeus anteriorly in males: not bilobate or bifurcated (0), bilobate or bifurcated (1).

Dorsum of body: minutely setose or glabrous (0), densely pubescent (1).

Elytron, longitudinal keels: no (0), 2 (1), 1 (2).

Phallobase: membranous ventro-proximally (0), tube shaped (1).

Phallobase ventrally: entirely membranous (0), sclerotised apically (1).

Phallobase, ventroapical sclerotization: 1 large sclerite (0), 2 swollen sclerites (1).

The complete list of the character states and the matrix are provided in the Suppl. materials 1, 2.

#### ﻿Tree topologies

**Figure 1.**
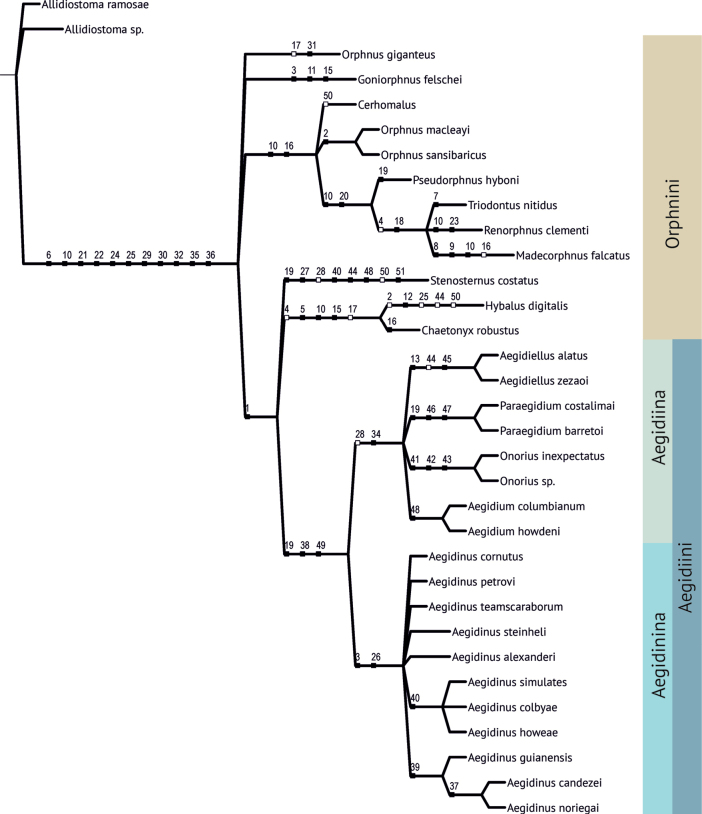
Phylogeny of Orphninae based on parsimony analysis of the revised character states from Frolov (Frolov 2012) with additional taxa. The strict consensus of six most parsimonious trees (83 steps, ci 84, ri 92). Black boxes – unique synapomorphies, white boxes – nonexclusive synapomorphies (homoplasies).

The parsimony phylogenetic analysis yielded six most parsimonious trees 83 steps long (Suppl. material 3). The trees show a similar topology differing mostly in the position of some Old World lineages. The strict consensus is shown in the Fig. 1.

### ﻿Taxonomical accounts

**Family Scarabaeidae Latreille, 1802**

**Subfamily Orphninae Erichson, 1847**

#### ﻿Tribe Aegidiini Paulian, 1984

##### ﻿Subtribe Aegidiina

Taxon classificationAnimaliaColeopteraScarabaeidae

Paulian, 1984

6F4EAC81-5D3F-5D72-87E3-64062B101700

###### Type genus.

*Aegidium*.

###### Diagnosis.

Small to medium-sized beetles (body length 5–20 mm), brown to black colored without pattern, more or less densely punctate, smooth or densely setose. Mandibles subsymmetrical, without lateral processes, distinctly or feebly protruding past anterior margin of frontoclypeus in dorsal view. Labrum exposed or hidden under clypeus in dorsal view. Frontoclypeus symmetrical or subsymmetrical, without tubercles, horns or ridges, or in males with variably shaped bilobate anterior frontoclypeal process. Pronotum of males may be with deep excavation in the middle, with 2 horns or ridges bordering the excavation near anterior margin (lateral pronotal processes), and with a tubercle or small horn medially on the anterior margin (anterior pronotal process); these characters are subject to allometric variability and may not be developed in some males. Females have a convex pronotum without armature or pronotum impressed anteriorly on disc and with a tubercle medially on anterior margin. Propleurae with carinae separating anterolateral areas from basal area. Scutellum narrowly rounded apically, about 1/8–1/13 length of elytra. Elytra convex, with marked humeral umbones (except for brachypterous species). Surface flat or with two low ridges in basal half; the ridges may be more or less convex, smooth, to almost indistinct. Pubescence of dorsal side indistinct or dense. Wings fully developed or vestigial. Metepisternon triangular, its posterior angle rounded to triangular and situated in distinct concavity of epipleuron. Mesocoxal cavities connected by a hole. Protibiae with three outer teeth, somewhat serrate basad of the teeth, with a smaller, medial tooth in majority of males. In males, anterior spur is absent. Each procoxae with one elongate hollow. Mesotibiae with or without a tuft of setae ventroapically in males. Stridulatory file with relatively fine, evenly spaced carinae. Phallobase tube shaped with strongly sclerotized ventral side but without differentiation of ventral and dorsal sclerites; ventroapical plate absent or present. Parameres symmetrical, relatively long, apices tapering or curved downwards, with or without setae; a few species have complex, feeble sclerotised processes on the parameres lateroapically. Endophallus without armature or with a small group of spinules; in one species of Aegidium there is a sclerite with two large curved spines. Spiculum gastrale T-, Y- or V-shaped, with setae on apical plate. Subcoxites oval, with dense, long setae mediabasally; coxites triangular, long, with dense short setae mediabasally and sparse long setae apically; stili distinct, elongated, or not separated from coxites.

###### Taxon composition.

The subtribe is comprised of *Aegidium* Westwood, 1845 (25 spp), *Paraegidium* Vulcano et al., 1966 (6 spp), *Aegidiellus* Paulian, 1984 (3 spp) and *Onorius* Frolov & Vaz-de-Mello, 2015 (2 spp).

###### Distribution.

Endemic to South and Central America.

##### ﻿ Aegidinina

Taxon classificationAnimaliaColeopteraScarabaeidae

Frolov, Akhmetova & Neita-Moreno subtr. nov.

A3507804-862F-5B85-92A1-F157682388D8

https://zoobank.org/CE492F83-9066-421E-B89B-53A36C1B1C4B

###### Type genus.

*Aegidinus* Arrow, 1904.

###### Diagnosis.

Body small to mid-sized (length 6 to 12 mm), reddish brown to dark brown. Mandibles subsymmetrical, with long processes on the outer sides. Clypeus with tubercle or horn on anterior margin medially in males, without horn in females. Pronotum variably excavated medially in males, convex to depressed medially in females; anterior margin of pronotum in males with a tubercle or horn medially. Propleura with carinae separating anterolateral areas from basal area. Scutellum narrowly rounded posteriorly, about 1/12 length of elytra. Elytra convex, with marked humeral umbones and striae marked with elongated punctures, surface smooth. Wings fully developed. Metepisternon triangular, its posterior angle rounded to triangular and situated in distinct concavity of epipleuron. Mesocoxal cavities not connected by a hole. Protibiae with three outer teeth, somewhat serrate basad of the teeth, with a smaller, medial tooth in majority of males. In males, anterior spur is absent. Each procoxa with two hollows. Mesotibiae without a tuft of setae ventroapically in males. Stridulatory file with wide carinae medially becoming much narrower and denser proximally. Phallobase tube shaped with strongly sclerotised ventral side but without differentiation of ventral and dorsal sclerites; ventroapical plate absent. Parameres relatively short, with complex shape but without feeble sclerotised processes, apices without setae; in some species parameres strongly asymmetric. Endophallus with relatively well-developed armature consisting of a few groups of spinules, sometimes of different size. Spiculum gastrale Y-shaped, without setae on apical plate. Subcoxites variably shaped, sometimes angulate or with a process mediabasally; coxites variably shaped, with armature sort robust spinules in some species mediabasally, stili distinct, variably shaped, or indistinct, not separated from coxites.

###### Taxon composition.

Only type genus, *Aegidinus* Arrow, 1904 (16 spp).

###### Distribution.

Endemic to South America.

##### ﻿ Aegidinus alexanderi

Taxon classificationAnimaliaColeopteraScarabaeidae

Frolov, Akhmetova & Neita-Moreno sp. nov.

2B5CDB80-B86F-58A4-AE5E-950218CDC3F6

https://zoobank.org/710F0E2D-0F47-4CB6-8355-2A71734E930E

Fig. 2A–H

###### Differential diagnosis.

**Figure 2.**
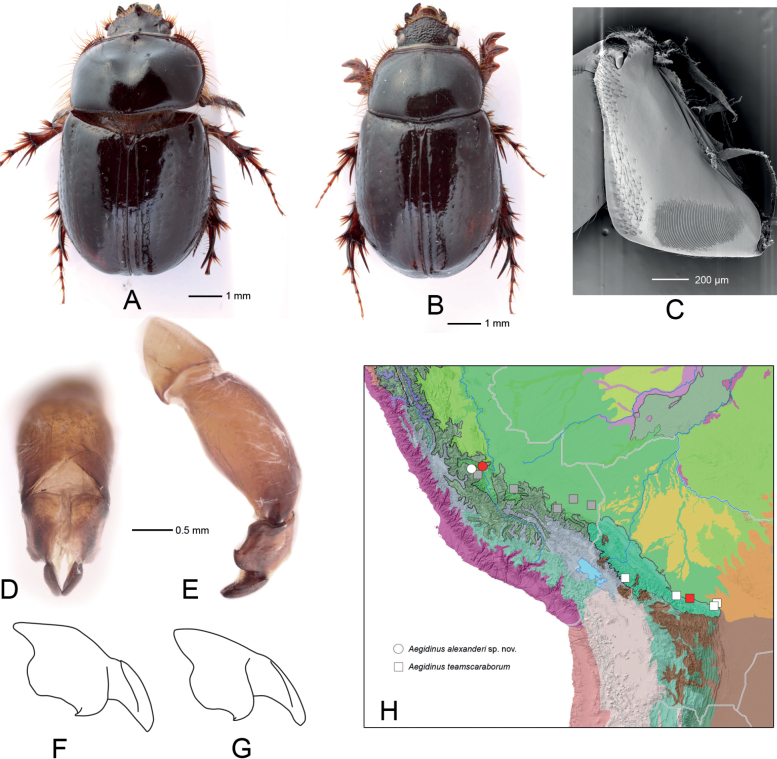
*Aegidinusalexanderi* sp. nov. (**A, D, E** male, holotype **B** female, paratype) and *A.teamscaraborum* (**G**) **A, B** habitus **C** stridulatory file, SEM **D** parameres in dorsal view **E** aedeagus in lateral view **F, G** paramere outline in lateral view (not to scale) **H** distributional record map (red symbols indicate holotype localities, gray squares indicate localities of *A.teamscaraborum* paratypes, which may belong to *A.alexanderi* sp. nov.).

*Aegidinusalexanderi* sp. nov. is most similar to *A.teamscaraborum* Colby, 2009, but differs from it in the shape of the parameres having proximal and distal lobes less separated and proximal lobes longer in lateral view (Fig. 2E–G). It should be noted that the differences between the two species are smaller than between most other *Aegidinus* species, implying their close relationships. It is possible that a thorough sampling in the Yungas will provide clear evidence of their allopatric or parapatric distribution and examination of molecular markers will show that the genetic distance between them is characteristic for subspecies rather than species; in this case, their status may be changed. Until such data are available, we suggest that these taxa are considered distinct species.

###### Type material.

***Holotype*.** Male at ZIN labeled “JUNÍN: Satipo Prov., 5 km NNE Puerto Ocopa, left bank of Perené River, near Canan Eden village, 1100 m a.s.l., vill. 8.III.2008. A.Petrov leg [FIDE6071]”. ***Paratypes*.** One male and one female [FIDE6072, FIDE6073] at CMN and two females [FIDE6074, FIDE6075] at ZIN with the same data as the holotype; one male and three females at ZIN labeled “PERU: Junin, 16 km NW Satipo, rio Venado, 1150 m 11°11.677'S, 74°46.137'W 13.III.2010 A. Petrov leg. [FIDE6076–FIDE6079]”.

###### Description.

**Male**, holotype (Fig. 2A, D, E).

***Body length*** 8.4 mm. Colour uniformly dark brown.

***Frontoclypeus*** wide, with convex anterior margin, slightly angulate laterally, somewhat crenulate. Genae small, slightly protruding past eyes. Frontal suture indistinct. Frontoclypeus with short conical horn rounded apically.

***Pronotum*** with widely rounded lateral margins, narrower than elytra, 1.6 times wider than length. Posterior angles widely rounded. Anterior margin bordered, border interrupted medially, with feeble gibbosity. Base of pronotum not bordered, with a few large rounded punctures laterally and a few small medially. Pronotal disc feebly excavated anteromedially, with two gibbosities in center. Pronotum punctate with a few large rounded punctures laterally and anteromedially and with minute, feebly visible punctures throughout.

***Scutellum*** subtriangular, narrowly rounded posteriorly, about 1/11 length of elytra.

***Elytra*** almost as long as wide, widest medially and rounded apically, with humeral and apical humps. First elytral stria as continuous line, connected basally with undulate line from scutellum to humeral hump. Other striae marked with rows of sparse punctures; punctures somewhat V- and comma-shaped on basal part of elytra, becoming smaller towards apices.

***Macropterous*.**

***Legs*.** Protibiae with 3 outer teeth, without medioapical tooth. Lateral margin basad of outer teeth not crenulate. Apical spur of protibia absent. Middle and hind legs similar in shape; metafemora and metatibiae about 1/8 longer than the mesofemora and mesotibiae. Mesotibia and metatibiae with 2 apical spurs, inner margin almost straight, outer margin with 1 transverse keel. Upper spur of hind tibiae as long as two basal tarsomeres. Claws 1/3 length of apical tarsomere. Femora almost impunctate.

***Abdomen*** ventrally irregularly punctate, pubescent, with sparse, long setae. Abdominal sternite 8 medially slightly longer than sternites 4–7 combined. Pygidium invisible from above, with slightly truncate apex in caudal view. Plectrum triangular with rounded apex, wider than long. Stridulatory file (Fig. 2C) with wide carinae medially becoming much narrower and denser proximally.

***Aedeagus*.** Phallobase without ventroapical plate. Parameres short (about 0.4 length of phallobase), curved downwards (Fig. 2D, E). Parameres with proximal lobes reasonably longer than sinuation between proximal and distal lobes in lateral view (Fig. 2E). Endophallus with 3 groups of spinules.

**Female** (Fig. 2B) differs from the male in having a relatively smaller pronotum without armature, frontoclypeus without process, and short but distinct protibial spur.

###### Paratypes and variability.

The body length of the examined specimens varies from 7.8–8.5 (males) and from 7.5–9.0 (females). Head and pronotal armature in one male paratype poorly developed with a small frontoclypeal tubercle and shallow pronotal fossa medially.

###### Distribution.

This species is known from two localities in Satipo Province in central Peru, mostly within the Peruvian Yungas ecoregion and on the border with Southwest Amazon moist forests ecoregion (Fig. 2H). The records of the paratypes *A.teamscaraborum* from the Peruvian Yungas (Fig. 2H, gray squares) are based on females only therefore may belong to *A.alexanderi* sp. nov. The holotype and other paratypes of *A.teamscaraborum* originate from Bolivian Yungas.

###### Etymology.

The new species is named after Alexander Petrov (Moscow) who collected and kindly donated us the specimens.

##### ﻿ Aegidinus elbae

Taxon classificationAnimaliaColeopteraScarabaeidae

Neita-Moreno, Akhmetova & Frolov sp. nov.

18FB8582-5948-5EE6-B195-111C1236F078

https://zoobank.org/89396271-30BC-4CA1-BC43-010C3871E8C3

Fig. 3A–E

###### Differential diagnosis.

**Figure 3.**
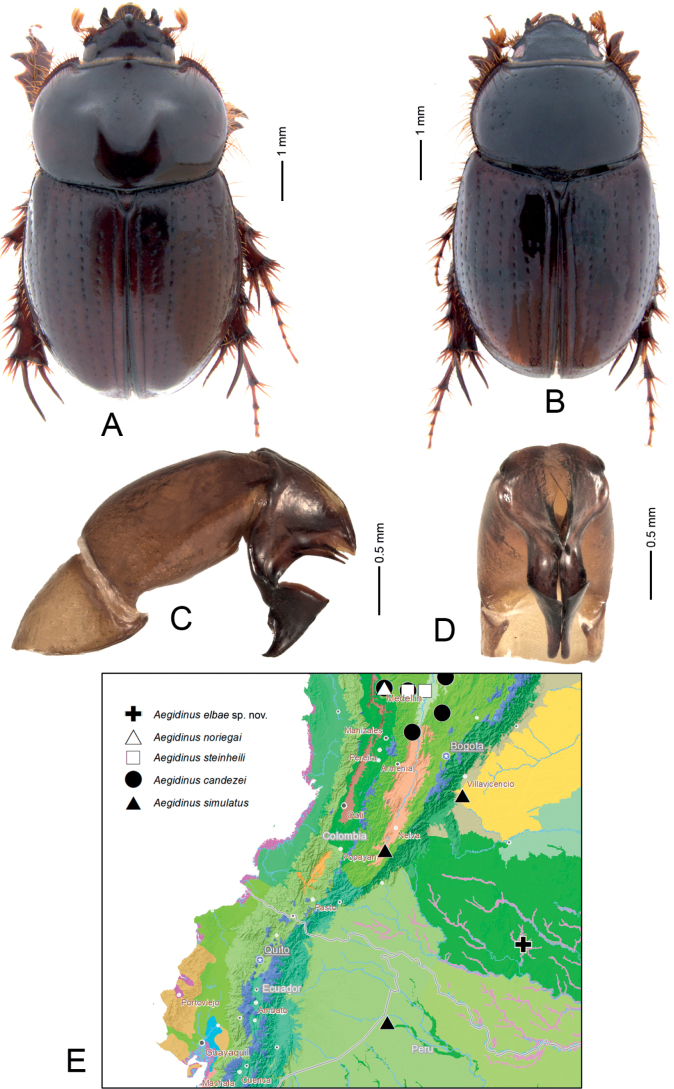
*Aegidinuselbae* Neita-Moreno, Akhmetova & Frolov sp. nov. (**A, C, D** male, holotype **B** female, paratype) **A, B** habitus **C** aedeagus in lateral view **D** parameres in dorsal view **E** distributional record map.

*Aegidinuselbae* sp. nov. is similar to *A.colbyae*Frolov et al., 2019, *A.brasiliensis* Arrow, 1904 and *A.howeae* Colby, 2009 in having mediobasal margins of dorsomedial lobes of parameres strongly sclerotized, protibia without medioapical tooth, and dorsal sides of parameres less overlapping, but differs from them in the parameres being distinctly longer and, in lateral view, abruptly separated into apical and basal parts (Fig. 3C).

###### Type material.

***Holotype*.** Male at IAvH (Fig. 3A): “COLOMBIA, Caquetá, Solano PNN/ Chiribiquete, Río Sararamano/Bosque Verde militar 300 m/0°14'47"N, 72°37'24"W pitfall T2/T1 3-5.iv.2000, E. Gonzalez Leg.”, “IAvH-E-256378”. ***Paratype*.** Female at IAvH (Fig. 3B) with following data: “COLOMBIA, Caquetá, Solano PNN/ Chiribiquete, Río Sararamano/Bosque Verde militar 300 m/0°14'47"N, 72°37'24"W pitfall /3-5.iv.2000, E. Gonzalez Leg.”, “IAvH-E-256379”.

###### Description.

Male (Fig. 3A, C, D).

***Body length*** 9.6 mm. ***Colour*** uniformly dark brown.

***Anterior*** margin of frontoclypeus with horn rounded apically.

***Pronotum*** with widely rounded lateral margins, narrower than elytra, 1.6 times wider than length. Posterior angles widely rounded. Anterior margin bordered, border complete medially, with feeble gibbosity. Base of pronotum not bordered, with a few large rounded punctures laterally and a few small medially. Pronotal disc feebly excavated anteromedially, with two gibbosities in centre. Pronotum punctate with a few large rounded punctures laterally and anteromedially and with minute, feebly visible punctures throughout.

***Scutellum*** subtriangular, narrowly rounded posteriorly, about 1/11 length of elytra.

***Elytra*** almost as long as wide, widest medially and rounded apically, with humeral and apical humps. First elytral stria as continuous line, connected basally with undulate line from scutellum to humeral hump. Other striae marked with rows of sparse punctures; punctures somewhat V- and comma-shaped on basal part of elytra, becoming smaller towards apices.

***Macropterous*.**

***Legs*.** Protibiae with 3 outer teeth, without medioapical tooth. Lateral margin basad of outer teeth not crenulate. Apical spur of protibia absent. Middle and hind legs similar in shape; metafemora and metatibiae about 1/8 longer than the mesofemora and mesotibiae. Mesotibia and metatibiae with 2 apical spurs, inner margin almost straight, outer margin with 1 transverse keel. Upper spur of hind tibiae as long as two basal tarsomeres. Claws 1/3 length of apical tarsomere. Femora almost impunctate.

***Aedeagus*.** Phallobase without ventroapical plate. Parameres long (about 0.7 length of phallobase). Parameres symmetrical, of complex shape (Fig. 3C, D): in the lateral view, they are abruptly separated into apical and basal parts; basal parts with 2 acute processes, apical parts somewhat dilating, giving the shape of a cup, acute and curved at very apex.

**Female** (Fig. 3B) differs from male in having relatively wider elytra, pronotum and head without excavations and armature, and in having a protibial spur. Body length 8.7 mm.

###### Distribution.

The species is known from a single locality in Caquetá, Colombian Amazonia (Fig. 3E).

###### Etymology.

The species is dedicated to Lic. Elba Moreno de Neita, mother of JCNM, to honor her memory.

### ﻿Key to the species of *Aegidinus* Arrow (males)

**Table:** 

1	Parameres separated into dorsomedial and ventrolateral lobes	**2**
–	Parameres not separated into dorsomedial and ventrolateral lobes	***Aegidinuscornutus* Colby, 2009**
2	Phallobase with ventroapical plate	**3**
–	Phallobase without ventroapical plate	**6**
3	Parameres symmetrical	**4**
–	Parameres asymmetrical	**5**
4	Ventrolateral lobe of paramere with subapical tooth	***Aegidinushowdenorum* Colby, 2009**
–	Ventrolateral lobe of paramere without subapical tooth	***Aegidinusguianensis* (Westwood, 1845)**
5	Parameres longer, more asymmetrical; ventroapical plate of phallobase longer than wide; protibia without medioapical tooth	***Aegidinusnoriegai* Frolov, Akhmetova & Vaz-de-Mello, 2019**
–	Parameres shorter, less asymmetrical; ventroapical plate of phallobase wider than long; protibia with medioapical tooth	***Aegidinuscandezei* (Preudhomme de Borre, 1886)**
6	Mediobasal margins of dorsomedial lobes of parameres feebly sclerotized, membranous; protibia with medioapical tooth	**7**
–	Mediobasal margins of dorsomedial lobes of parameres strongly sclerotized; protibia without medioapical tooth	**10**
7	Ventrolateral lobes of parameres long and slender (in lateral view), reasonably longer than dorsomedial lobes	***Aegidinussteinheili* (Harold, 1880)**
–	Ventrolateral lobes of parameres triangular and obtuse in lateral view, not longer than dorsomedial lobes	**8**
8	Ventrolateral lobes of parameres as long as dorsomedial lobes	***Aegidinuspetrovi* Colby, 2009**
–	Ventrolateral lobes of parameres reasonably shorter than dorsomedial lobes	**9**
9	Parameres with proximal and distal lobes more separated and proximal lobes shorter in lateral view	***Aegidinusteamscaraborum* Colby, 2009**
–	Parameres with proximal and distal lobes less separated and proximal lobes longer in lateral view (Fig. 2E)	***Aegidinusalexanderi* Frolov, Akhmetova & Neita-Moreno, sp. nov.**
10	Dorsal sides of parameres strongly overlapping and separated by slit	***Aegidinussimulates* Colby, 2009**
–	Dorsal sides of parameres less overlapping and not separated by slit	**11**
11	In lateral view, parameres longer (about 0.7 length of phallobase) and abruptly separated into apical and basal parts (Fig. 3C)	***Aegidinuselbae* Neita-Moreno, Akhmetova & Frolov, sp. nov.**
–	In lateral view, parameres shorter (about 0.4 length of phallobase), not abruptly separated into apical and basal parts	**12**
12	Dorsal processes of parameres carina-shaped	***Aegidinuscolbyae* Frolov, Akhmetova & Vaz-de-Mello, 2019**
–	Dorsal processes of parameres tooth or spur-shaped	**13**
13	Dorsal processes of parameres long, spur-shaped	***Aegidinusbrasiliensis* Arrow, 1904**
–	Dorsal processes of parameres short, tooth-shaped	***Aegidinushoweae* Colby, 2009**

## ﻿Discussion

Although the monophyly of Aegidiini was not questioned (Frolov 2012), the phylogenetic relations of the genera comprised the tribe were unclear (Colby 2009, Frolov et al. 2019). Colby (2009) presented two consensus trees, one based on a full set of taxa she studied and another one with *Stenosternus* Karsh, 1881 and *Goniorphnus* Arrow, 1911, excluded. The first tree shows polytomy in respect to the Aegidiini and most other taxa. The second tree resolved Aegidiini as a separate clade and showed *Aegidinus* as a sister taxon for *Aegidium*. The two genera, in turn, were sister to *Aegidiellus*, and all three – to *Paraegidium*. All these clades have low bootstrap support and were based, apparently, on homoplastic characters. In Frolov’s (2012) analysis, the clade (*Aegidinus* + *Aegidiellus* + *Paraegidium*) was shown to be sister to *Aegidium*. However, this clade was supported by the single homoplasy, the absence of the protibial process in males. Later, after examination of all *Aegidinus* species, it was found that the process is present in some of them (Frolov et al. 2019).

The analysis reported here, based on the expanded and verified set of characters and all nominal supraspecific Orphninae taxa, provides strong support for the two lineages of the Aegidiini. One lineage includes *Aegidium* and three related genera. It has a unique synapomorphy, the hole connecting mesocoxal cavities, and a non-unique synapomorphy, the absence of the transverse keel on hind tibiae, shared with *Stenosternus*. However, the latter state could be gained independently by the New World and Old World taxa. The second lineage included the genus *Aegidinus* and is characterised by two unique synapomorphies, mandibles with long processes on the outer sides and two procoxal hollows.

To make the classification better reflect the phylogenetic relations of the taxa in questions, and specifically to distinguish the *Aegidium* group lineage as a separate taxon we introduced a subtribal system for Aegidiini. The nominotypical subtribe, Aegidiina, includes *Aegidium*, *Paraegidium*, *Aegidiellus* and *Onorius*, and a new monotypical subtribe, Aegidinina subtr. nov. is erected to accommodate *Aegidinus*.

*Aegidinus* now comprises 15 species and is the second most species-rich genus of the South American Orphninae, after *Aegidium*. The bionomy of its species is virtually unknown and almost all species were recorded from a small series of specimens. It is possible that more species are yet to be described, specifically from the Andes, which is apparently the centre of diversity and diversification of the genus. Our results also suggest that the *Aegidinus* is composed of a few lineages, which may necessitate establishing subgeneric or species group classification, however the analysis of the phylogenetic relations of the taxa at species level is outside the scope of the present contribution.

## Supplementary Material

XML Treatment for Aegidiina

XML Treatment for Aegidinina

XML Treatment for Aegidinus alexanderi

XML Treatment for Aegidinus elbae

## ﻿Additional information

### Author ORCIDs

Andrey V. Frolov https://orcid.org/0000-0002-6724-6828

Lilia A. Akhmetova https://orcid.org/0000-0002-2151-1278

Jhon César Neita-Moreno https://orcid.org/0000-0003-2998-2063

### Data availability

All of the data that support the findings of this study are available in the main text or Supplementary Information.

## Supplementary materials

Supplementary material 1 Character states Data type morphological (word document) Explanation note Morphological character states for phylogenetic analysis. https://binary.pensoft.net/file/860271This dataset is made available under the Open Database License (http://opendatacommons.org/licenses/odbl/1.0/). The Open Database License (ODbL) is a license agreement intended to allow users to freely share, modify, and use this Dataset while maintaining this same freedom for others, provided that the original source and author(s) are credited.Andrey V. Frolov, Lilia A. Akhmetova, Jhon César Neita-Moreno

Supplementary material 2 Character matrix Data type phylogenetic (word document) https://binary.pensoft.net/file/860272This dataset is made available under the Open Database License (http://opendatacommons.org/licenses/odbl/1.0/). The Open Database License (ODbL) is a license agreement intended to allow users to freely share, modify, and use this Dataset while maintaining this same freedom for others, provided that the original source and author(s) are credited.Andrey V. Frolov, Lilia A. Akhmetova, Jhon César Neita-Moreno

Supplementary material 3 Phylogenetic trees Data type images (PDF file) Explanation note Six most parsimonious trees, resulted from phylogenetic analysis. https://binary.pensoft.net/file/860273This dataset is made available under the Open Database License (http://opendatacommons.org/licenses/odbl/1.0/). The Open Database License (ODbL) is a license agreement intended to allow users to freely share, modify, and use this Dataset while maintaining this same freedom for others, provided that the original source and author(s) are credited.Andrey V. Frolov, Lilia A. Akhmetova, Jhon César Neita-Moreno
