# Supplementary material for: ﻿Phylogenetic analysis of the Neotropical scarab beetle tribe Aegidiini (Coleoptera, Scarabaeidae, Orphninae) with description of new taxa
Source: Zookeys. 2023 Jun 6;1166:33–47. doi: 10.3897/zookeys.1166.102813 (PMC10265217; doi:10.3897/zookeys.1166.102813)
Supplement: Supplementary material 1 — Character states [file zookeys-1166-033_article-102813__-s001.docx]

**Supplementary materials. Character states.**

0. Mandible without carina ventrally (0), with carina (1).

1. Number of mandibular teeth medially: 2 (0), 3 (1), 4 (2), 1 (3).

2. Shape of mandible external margin: rounded (0), angulate (1), with a process (2).

3. Mandibles: symmetrical or subsymmetrical (0), asymmetrical (1).

4. Mandible apex: not widened sagitally (0), widened sagitally (1).

5. Mandible molar area: well developed (0), poorly developed (1).

6. Left mandible molar area: appears entire (0), strongly serrate (1).

7. Right mandible in male: subequal to left (0), considerably longer (1).

8. Lacinia: with strongly sclerotized apex and 1 spinule basally (0), long apex without spinules surrounding (2), with bi- or trifurcate apex (3), with short apex and fine setation basally (5).

9. Galea: with spinelike apex, robust spinule nearby and a brush of long seta (0), without distinct apex, some setae in brush stronger (1), with fingershape apex and 1-2 robust spinules (2), with feeble distinct apex and 2 robust setae (3), with feeble distinct apex and brus (4), with indistinct apex and fine brush (6)

10. Second labial palpomere: without angulate process directed mediad (0), with angulate process mediad (1)

11. First segment of antennal club: square to 7th antennomere (0), inclined to 7th antennomere (1)

12. First segment of antennal club: glabrose (0), sparcely setose, mostly apically (1), entirely densely setose (2)

13. Labrum, fore margin: more or less bilobate (0), protruding megially (1), somewhat straight or very feebly sinuate (2)

14. Labrum, shape of basal circular structure: rounded or subtriangular (0), heart shaped (1), wide, suboval to subtriangular (2), subtriangular with feebly concave anterior margin (3)

15. Labrum, apical circular structure: more or less distinct (0), indistinct or absent (1), with short processes (2)

16. Labrum, longitudinal line in basal circular structure: more or less distinct or reaching base (0), indistinct or absent (1)

17. Labrum, apical circular structure: much smaller than the main, not reaching the fore margin (0), almost as large as the main, nearly reaching the margin (1)

18. Elytral sculpture: with round punctures (0), V-shaped proximad (1), with large elongate punctures, paler than elytra (2), with semicircular punctures (3), with U-shaped punctures directed caudad (4), with U-shaped punctures directed proximad (5)

19. Elytron, basal border: absent (0), present (1)

20. Elytron, basal slope: somewhat convex (0), large and concave, with border separating from disc (1)

21. Fore tibia spur: present in both sexes (0), absent in males (1)

22. Apical outer tooth of fore tibia: directed at right or obtuse angle to inner margin (0), directed parallel with inner margin (1)

23. Fore tibia apical setae: slender, as other on inner margin (0), thicker (normally 3) (1)

24. Cavity (furrow) on anterior coxa: absent (0), present (1)

25. Number of procoxal cavities: one (1), two (2)

26. Middle tibiae transverse carina: absent (0), present (1)

27. Hind tibia transverse carina: absent (0), present (1)

28. Stridulatory file: as numerous longitudinal carinae (0), as one transverse ridge (1)

29. Triangular plectrum on first abdominal sternite: absent (0), present (1)

30. Longitudinal ridges of stridulatory file: fine and evenly spaced (0), middle are coarser and more widely spased (1)

31. Distance between tibial spur arcticulations: about the same in hind and middle legs (0), much smaller in middle legs, spurs almost adjoining (1)

32. Metepisternon: not widened posteriorly (0), widened posteriorly to lock closed elytra (1)

33. Middle coxal cavities: not connected (0), connected by a hole (1)

34. Mandibular gland duct: absent (0), present (1)

35. Bursa copulatrix: membranous (0), sclerotized (1)

36. Parameres: symmetrical (0), asymmetrical (1)

37. Stridulatory ridges: straight (0), distinctly curved posteriad (1)

38. Phallobase protruding ventroapical plate: absent (0), present (1)

39. Mediobasal margins of parameres: feebly sclerotised (0), strongly sclerotised (1), strongly sclerotised and serrate (2)

40. Mandibles visible from above: yes (0), no or feebly (1)

41. Labrum visible from above: yes (0), no (1)

42. Tarsi: slender (0), robust (1)

43. Paramere apices: glabrous (0), with short setation (1), with long setation (2)

44. Tubercle on anterior margin of pronotum in female: absent (0), present (1)

45. Clypeus anteriorly in males: not bilobate or bifurcated (0), bilobate or bifurcated (1)

46. Dorsum of body: minutely setose or glabrous (0), densely pubescent (1)

47. Elytron, longitudinal keels: no (0), 2 (1), 1 (2)

48. Phallobase: membranous ventro-proximally (0), tube-shaped (1)

49. Phallobase ventrally: entirely membranous (0), sclerotised apically (1)

50. Phallobase, ventroapical sclerotization: 1 large sclerite (0), 2 swollen sclerites (1)
