# Supplementary material for: ﻿Phylogenetic analysis of the Neotropical scarab beetle tribe Aegidiini (Coleoptera, Scarabaeidae, Orphninae) with description of new taxa
Source: Zookeys. 2023 Jun 6;1166:33–47. doi: 10.3897/zookeys.1166.102813 (PMC10265217; doi:10.3897/zookeys.1166.102813)
Supplement: Supplementary material 2 — Character matrix [file zookeys-1166-033_article-102813__-s002.docx]

**Supplementary materials. Table 1. Character matrix**

0 5 10 15 20 25 30 35 40 45 50

| | | | | | | | | | |

Allidiostoma ramosae 03000100560011-1--0000000-1110-0001100000000000000-

Allidiostoma sp. 03000100560011-1--0000000-1110-0001100000000000000-

Orphnus macleayi 02000000000010000000110111110101000000000000000000-

Cerhomalus 010000000000100000001101111101010000000000000000011

Orphnus sansibaricus 02000000000010000000110111110101000000000000000000-

Orphnus giganteus 01000000010010011-00110111110111000000000000000000-

Stenosternus costatus 10000000310012010-40110-110001011000000200020002010

Paraegidium costalimai 10000000310011010-50110111100101110001000000011010-

Paraegidium barretoi 10000000310011010-50110111100101110001000000011010-

Aegidium columbianum 10000000310021010-20110111100101110001000000000110-

Aegidium howdeni 10000000310021010-20110111100101110001000000000110-

Onorius inexpectatus 10000000310021010-00110111100101110001001110000010-

Onorius sp. 10000000310021010-10110111100101110001001110000010-

Aegidinus simulates 10200000310011010-20110112110101100001010000000010-

Aegidinus colbyae 10200000310011010-20110112110101100001010000000010-

Aegidinus howeae 10200000310011010-20110112110101100001010000000010-

Aegidinus steinheli 10200000310011010-20110112110101100001000000000010-

Aegidinus cornutus 10200000310011010-20110112110101100001000000000010-

Aegidinus guianensis 10200000310011010-20110112110101100001100000000010-

Aegidinus candezei 10200000310011010-20110112110101100011100000000010-

Aegidinus noriegai 10200000310011010-20110112110101100011100000000010-

Aegidinus petrovi 10200000310011010-20110112110101100001000000000010-

Aegidinus teamscaraborum 10200000310011010-20110112110101100001000000000010-

Aegidinus alexanderi 10200000310011010-20110112110101100001000000000010-

Aegidiellus alatus 10000000310001010-20110111100101110001000001100010-

Aegidiellus zezaoi 10000000310001010-20110111100101110001000001100010-

Goniorphnus felschei 01100000011010110-00110111110101000000000000000000-

Hybalus digitalis 11011000320112311-0011010-1101010000000000010000011

Chaetonyx robustus 10011000320012321-00110111110101000000000000000000-

Pseudorphnus hyboni 01000000030010000031110111110101000000000000000000-

Triodontus nitidus 01010010030010000101110111110101000000000000000000-

Renorphnus clementi 01010000040010000101111111110101000000000000000000-

Madecorphnus falcatus 01010001250010010-01110111110101000000000000000000-
