# Supplementary figures and images for: ﻿Phylogenetic analysis of the Neotropical scarab beetle tribe Aegidiini (Coleoptera, Scarabaeidae, Orphninae) with description of new taxa
Source: Zookeys. 2023 Jun 6;1166:33–47. doi: 10.3897/zookeys.1166.102813 (PMC10265217; doi:10.3897/zookeys.1166.102813)

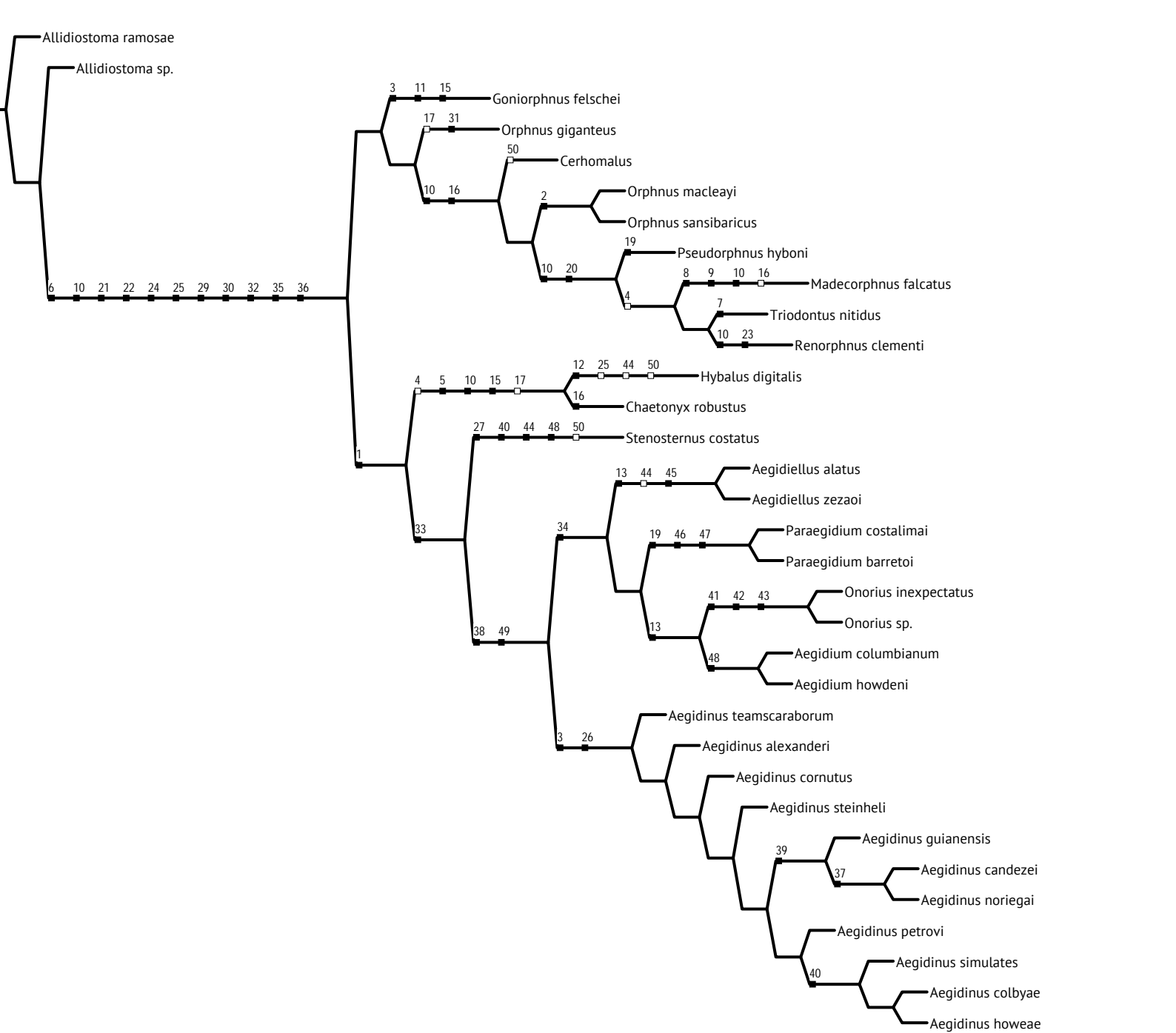

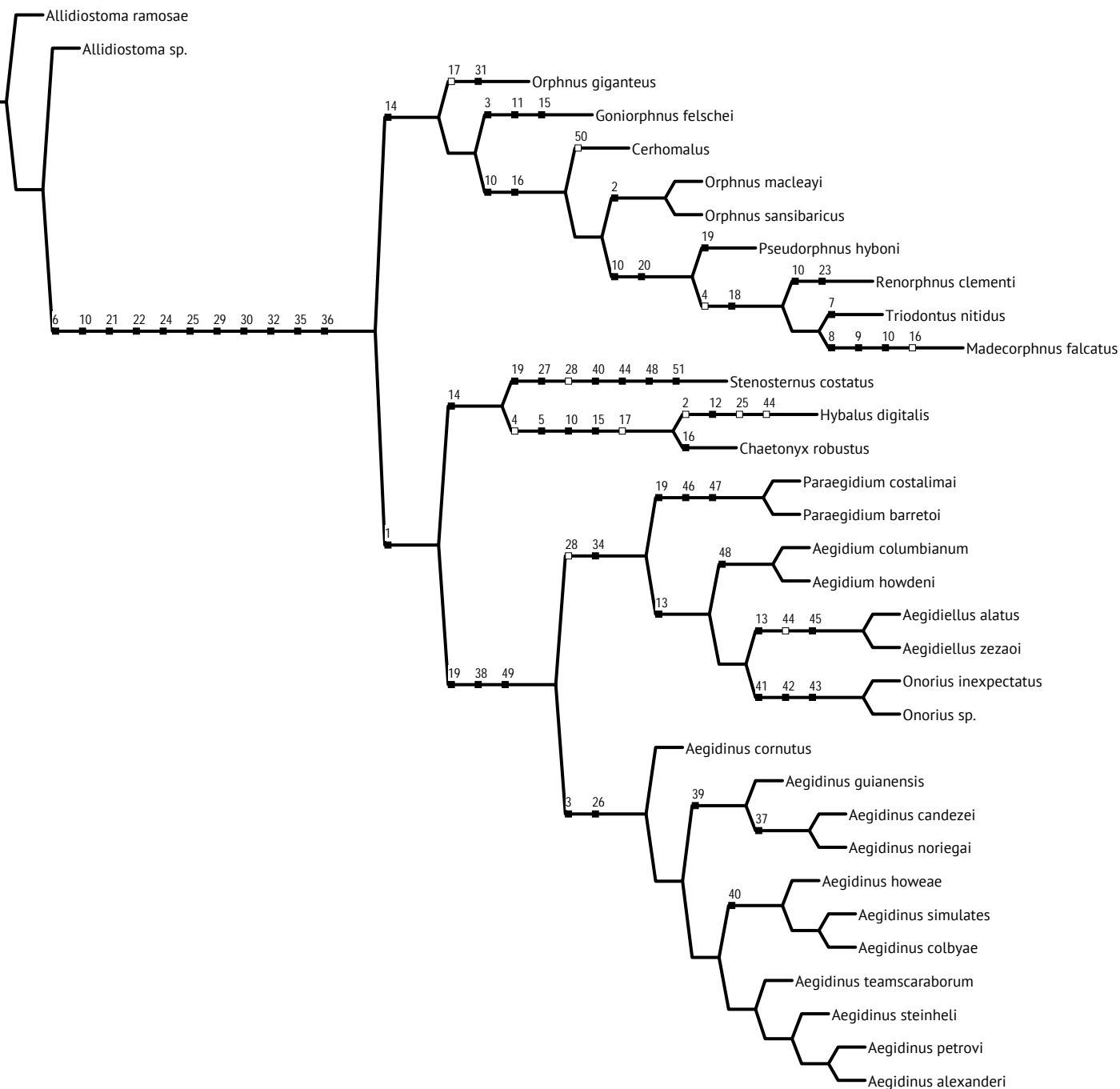

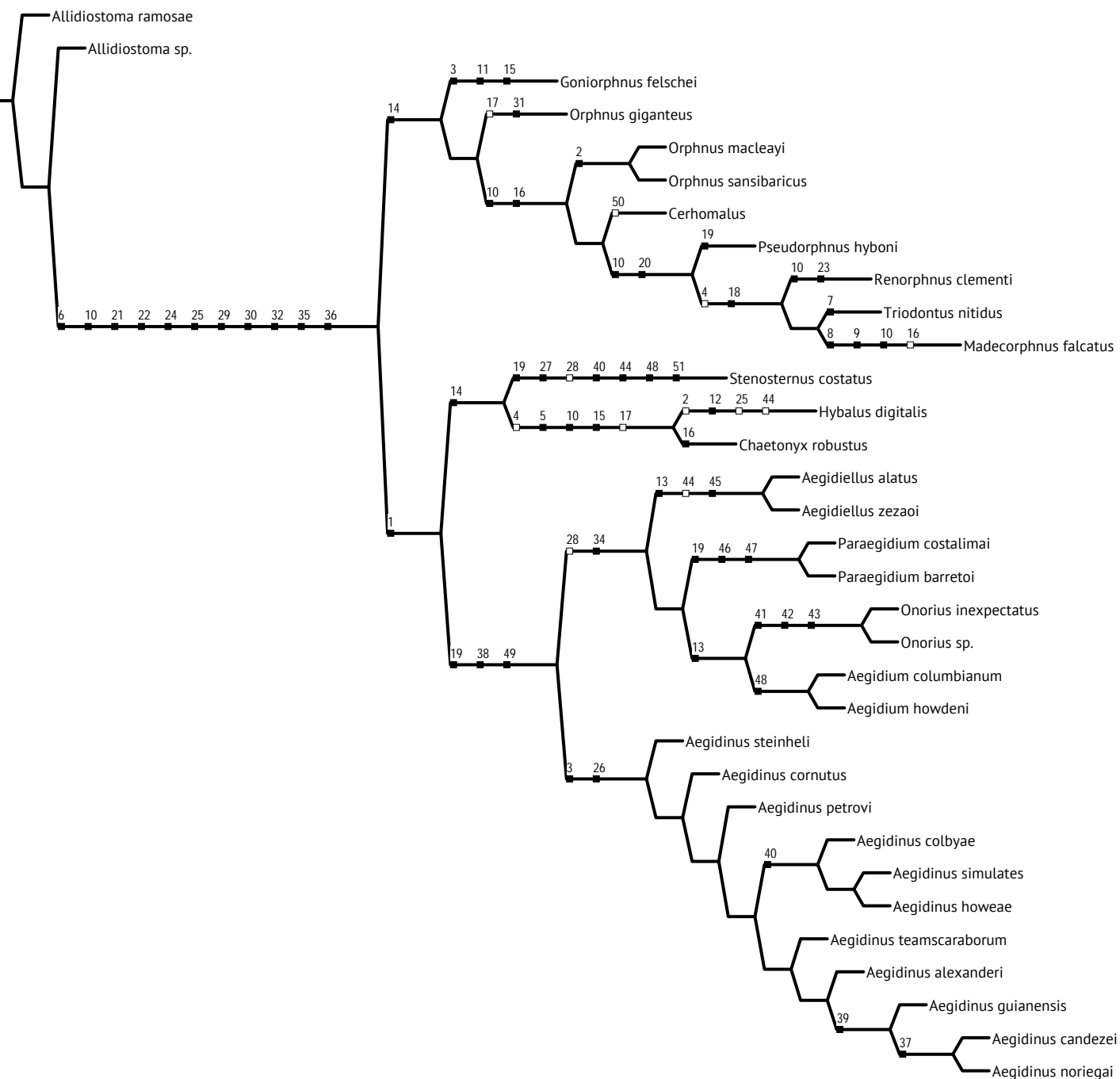

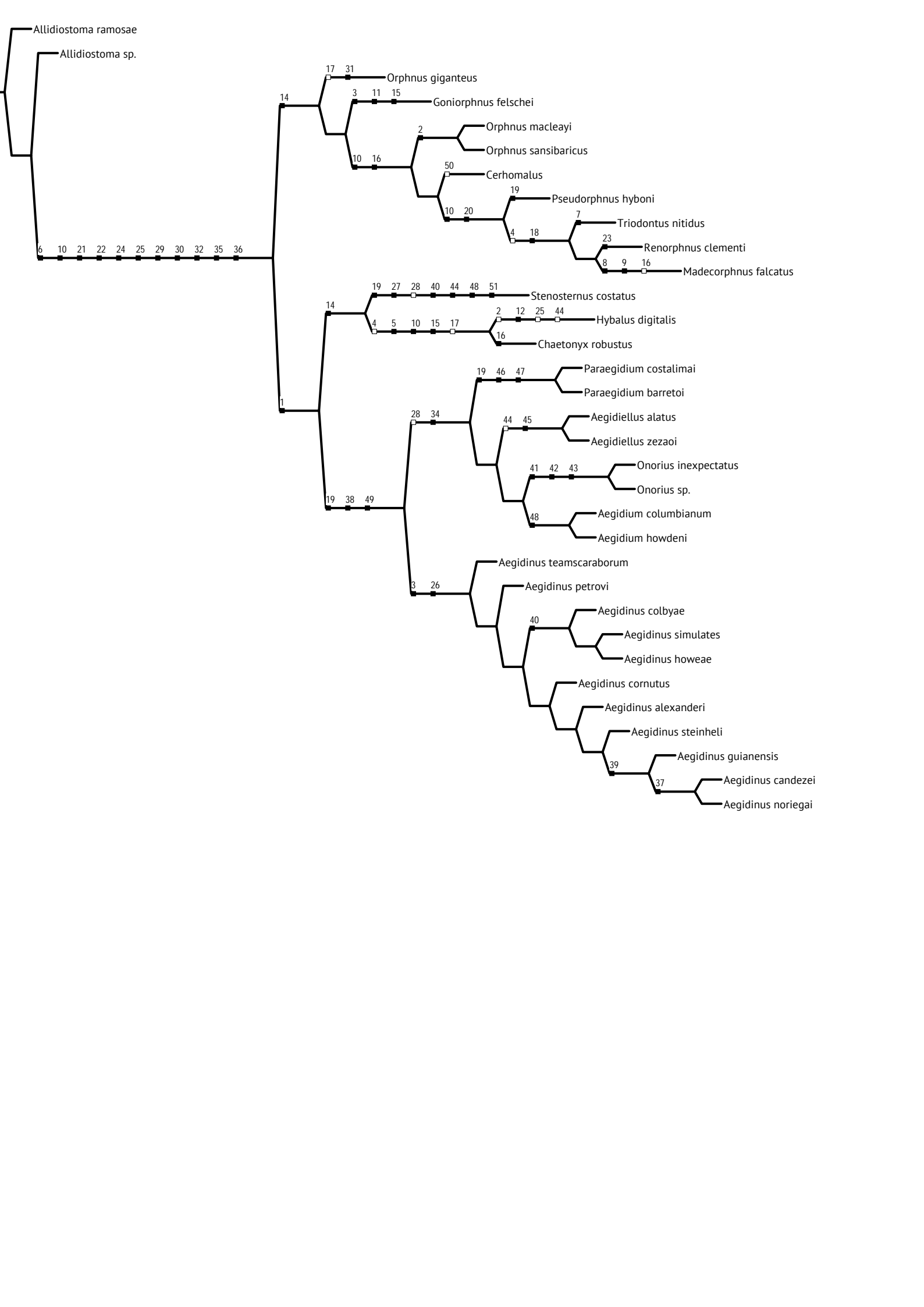

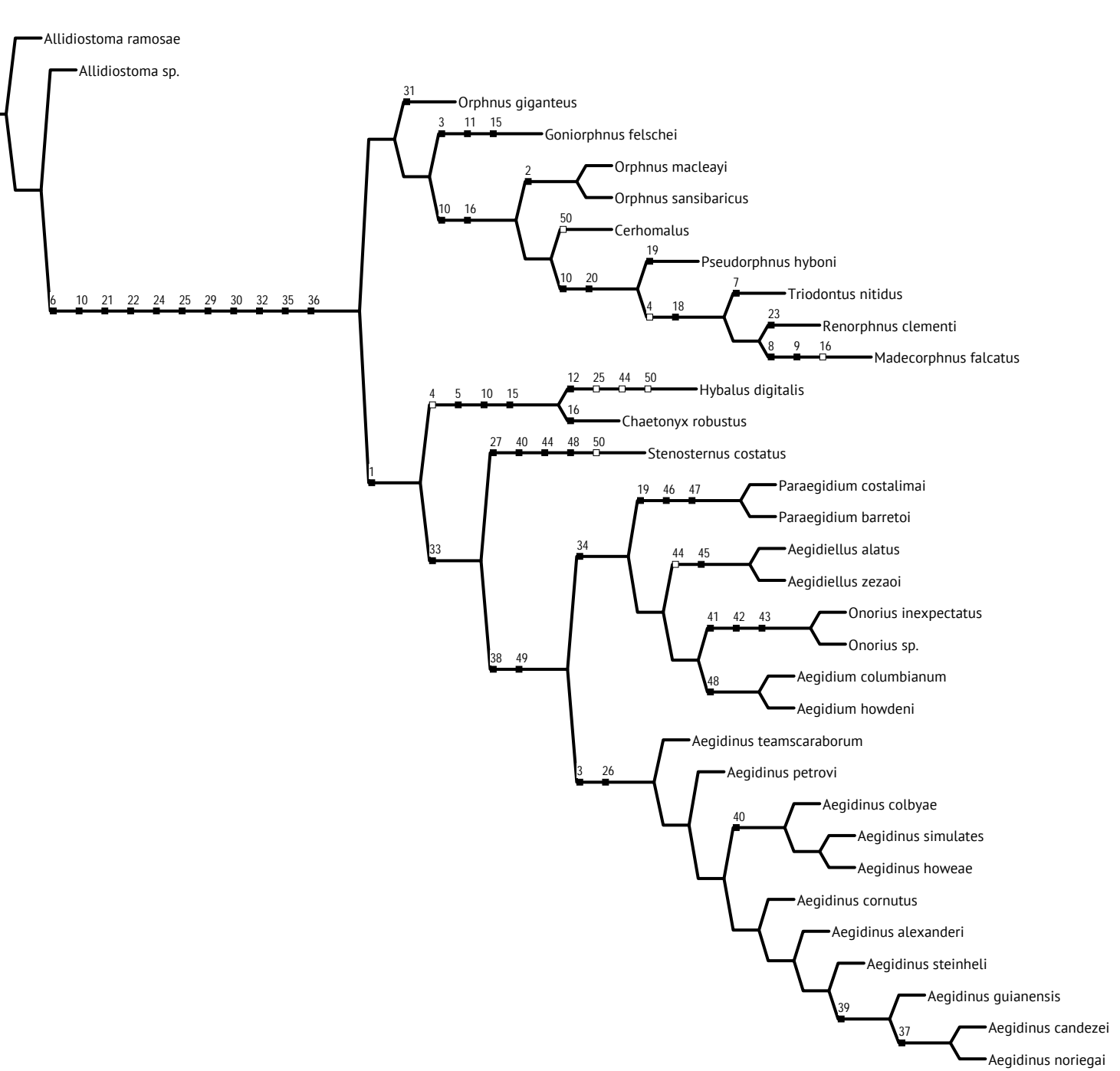

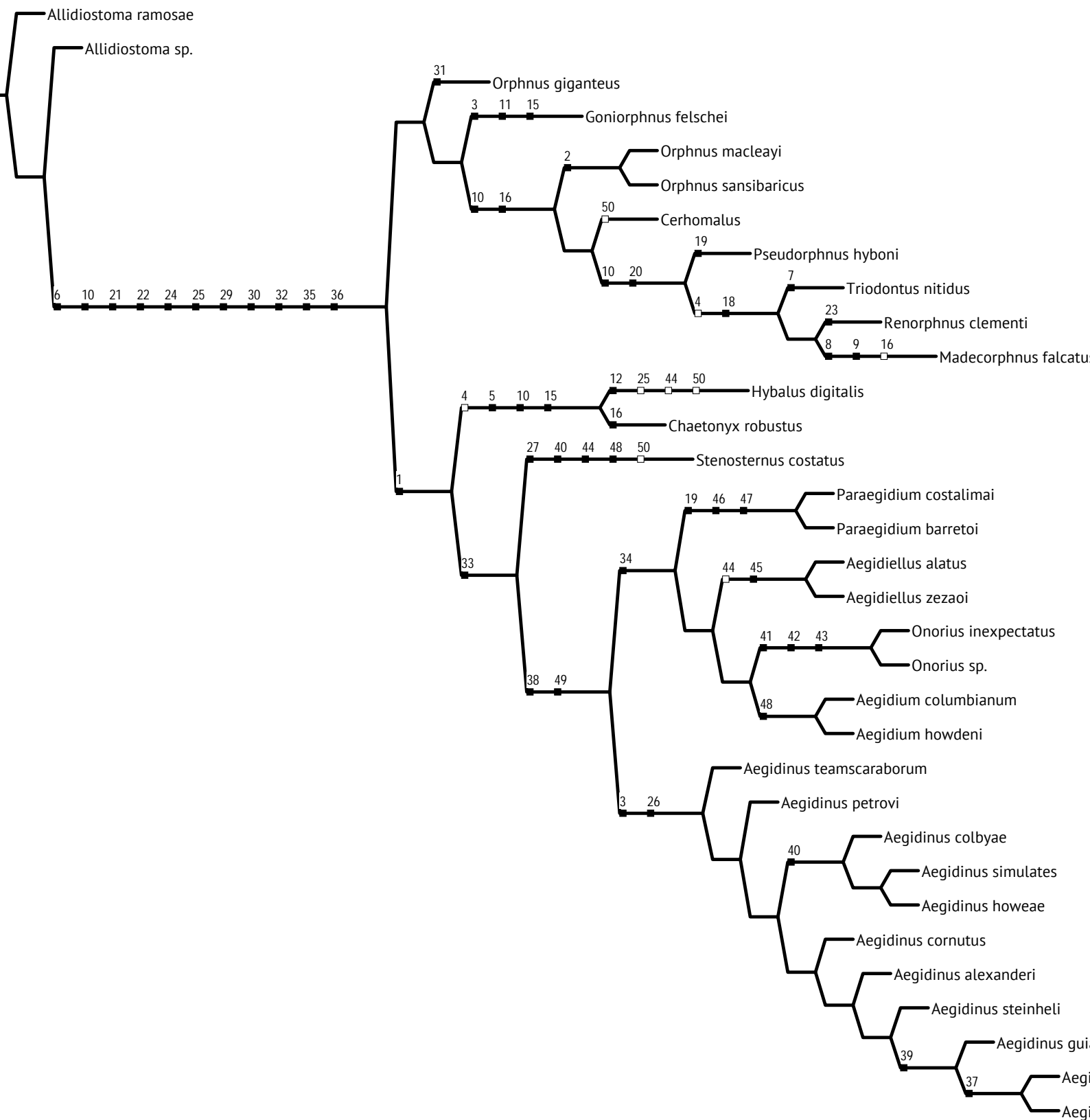

Supplement: Supplementary material 3 — Phylogenetic trees [file zookeys-1166-033_article-102813__-s003.pdf]
